# American Resorts, with Notes upon Their Climate

**Published:** 1890-03

**Authors:** 


*American Resorts, with Notes upon their Climate.* By BUSHROD W. JAMES, A.M., M.D. Pp. 285. Philadelphia and London: F. A. DAVIS. 1889.

This is a well-got-up and thoroughly practical work on American Health-resorts, in which is included a translation of Werkof's work on *Climatology*, so far as it relates to America and contiguous islands. That America has, within its own limits, an almost endless variety of climate suitable for invalids is an admitted and well-known fact. This work will serve as an excellent guide to the selection of a resort for health.

Thousands of English readers are familiar with American authors in magazines, novels, poems and philosophy. The American *can* write clear and pure English; that is abundantly evident. The medical American writer does not always do so; in this case he does not. "Locate" and "Location," for instance, occur on almost every page: these words are not employed by cultured writers of English.

